# Constructing the World and Locating Oneself

**DOI:** 10.1007/s13164-017-0357-0

**Published:** 2017-08-31

**Authors:** Peter Pagin

**Affiliations:** Department of Philosophy, Stockholm University, Stockholm, Sweden

## Abstract

In *Our Knowledge of the Internal World*, Robert Stalnaker describes two opposed perspectives on the relation between the internal and the external. According to one, the internal world is taken as given and the external world as problematic, and according to the other, the external world is taken as given and the internal world as problematic. Analytic philosophy moved from the former to the latter, from problems of world-construction to problems of self-locating beliefs. I argue in this paper that these problems are equivalent: both arise because experience and objective, external facts jointly underdetermine their relation. Both can be seen as a problem of expressive completeness; of the internal language in the former case, and of the non-indexical language in the second.

## Introduction

In the beginning of *Our Knowledge of the Internal World*, Robert Stalnaker characterizes the difference between two perspectives on reality, the *internalist* and the *externalist*. Stalnaker writes:

The Cartesian internalist begins with the contents of his mind—with what he finds by introspecting and reflecting. This is what is unproblematic; these are the things and the facts we know directly. The internalist’s problem is, how do we move beyond these to form a conception of an external world, and how are we able to know that the world beyond us answers to the conceptions that we form. The externalist, in contrast, proposes that we begin with the world we find ourselves in, and with what either common sense or our best scientific theories tell us about it. Among the things we find are human beings—ourselves—who are things that (it seems) can know about the world, can experience it, have apoint of view on it. Our problem is to explain how our objective conception of the world can be a conception of aworld that contains things like us who are able to think about and experience it in the way that we do (Stalnaker 2008, pp. 2–3).

In short: the internalist takes subjective internal facts as basic/given and objective external facts as problematic, while the externalist takes objective external facts as basic/given and subjective internal facts as problematic.^1^

Stalnaker takes Hume and Kripke as examples of an internalist and an externalist, respectively. Interestingly, we can view the history of the past hundred years of (relevant parts of) analytic philosophy as moving from the internalist perspective to the externalist perspective, from regarding the physical world as problematic to regarding the internal world as problematic.

It is natural to regard the two grand world-constructing projects of the early 20th century as representing the internalist perspective. These are Russell’s *Our Knowledge of the External World* (Russell 1914) and Carnap’s *Der Logische Aufbau der Welt* (Carnap 1928). Both Russell and Carnap attempted to *construct* the physical world on the basis of what is immediately given in the sense of *reducing* statements that appear to be about the physical world to statements that are not. The reduction in both Russell and Carnap takes place by means of the formal apparatus of Russell’s and Whitehead’s *Principia Mathematica* (Whitehead and Bertrand Russell 1910).

In the respect of being internalists, there are substantial differences between Russell and Carnap. Russell appealed to *sense-data*, that which is immediately given to the senses (Russell 1914, p. 35). The constructing subject uses his/her own sense data. The sense-data are not considered as internal to a mind, only as those parts of reality that are directly given to a mind. Russell further appealed to *possible sense-data*, which become actual when experienced but can remain unactualized.

Compared with Russell, Carnap was a more thorough-going internalist. The empirical basis for Carnap is explicitly mental (“auto-psychological”, experienced by the constructing subject). In fact, Carnap uses only one single basic concept, the relational concept of a *recollection of similarity* (*Ähnlichkeitserinnerung*) between elementary experiences (Carnap 1928, p. vii). Both Russell and Carnap had the idea that the basic language in which the construction is carried out is *expressively complete*. By definition, every proposition (if it can be expressed at all) can be expressed in an expressively complete language. Some propositions may require very complicated sentences to express, but clever definitions can allow us to express them in a concise way.^2^

Not long after the publication of the *Aufbau*, however, Carnap was convinced by Otto Neurath that the requirement of the intersubjectivity of science could not be met by a construction from a mental basis.^3^ He made the move, in ‘Die Physicalische Sprache als Universalsprache derWissenschaft’, of switching from the psychological to the *physicalistic* language as the basic language. The basic language was then conceived of as *universal* in the sense that every (meaningful) sentence can be translated into a sentence of that language (Carnap 1932, p. 437). The most elementary sentences of the physicalistic language were the *protocol sentences* (Carnap 1932, pp. 438-39). Carnap offers three alternative types of protocol sentence, without himself wanting to choose between them. The alternatives were

1.
  - ‘Now joy’ (‘Jetzt Freude’); ‘Now here blue, there red’ (‘Jetzt hier Blau, dort Rot’.)
  - ‘Now red circle’ (‘Jetzt roter Kreis’).
  - ‘A red die is lying on the table’ (‘Auf dem Tisch liegt ein roter Würfel’).

The choice between these types of protocol sentence had been a matter of debate between the members of the Vienna Circle, but these differences need not concern us here. Two things are to be noted, however.

The first is the universal character of the language: that any (meaningful) sentence can be translated into the physicalistic language has the consequence that the physicalistic language is *expressively complete*: all possible states of affairs can be expressed in it. In fact, Carnap argues that psychological sentences can be translated into physicalistic sentences by means of empirical correlations of states. The sentence

(2) *S*’s body is now red-seeing

which is an abbreviated description of a state of *S*’s body, is correlated with the sentences

(3)
  - *S* is now seeing red
  - Now red

where (3a) and (3b) are protocol sentences (Carnap 1932, p. 457). The correlation consists in the fact that the physical state can be identified by certain stimulus-response dispositions, including the disposition to utter a sentence like (3b) upon being shown something red.

The appearance that (2) has a different meaning from (3a) is dismissed as a mistake generated from the misleading *material form* of speech. In the formal mode of speech, there is nothing more to sameness of meaning than *interderivability*. The interderivability of (2) and (3a) is established by means of the empirical correlation itself (Carnap 1932, p. 458). Thanks to this correlation, the protocol language of a person *S* becomes a part of the physicalistic language.

We can see here that Carnap himself switched over from an internalist to an externalist perspective around 1930. The problem of reducing sentences of a physicalistic language to an experience language is replaced by the problem of incorporating the protocol language into the physicalistic language by means of translation.

The second thing to note is that Carnap explicitly allows the use of indexicals in protocol sentences. There is no hint in Carnap (1932) that it is possible, or desirable, to replace indexicals by constant terms. In hindsight, we can see a clear connection between the reduction project and the elimination of indexicals. That indexicals are eliminable means that any proposition that can be expressed in language *L* with indexicals can be expressed in a language *L*′ free of indexical context dependence. In particular, this holds if *L*′ is expressively complete.

Carnap (1936) presents a new reason why the reduction project can not succeed: theoretical sentences cannot be reduced to protocol sentences (observation sentences) in the sense that for each theoretical sentence there is an equivalent sentence of the protocol language.^4^ Although a theoretical sentence entails sentences of the protocol language, it is not entailed by any finite set of protocol sentences. Carnap illustrates this with the example of solubility:

(4)
  - *x* is soluble in water.
  - Whenever *x* is put into water, *x* dissolves.

Carnap (1936, p. 440). Carnap notes that (4b) does not mean the same as (4a), for (4b) is automatically true of an object that is never put into water, while (4a) isn’t.^5^ Carnap instead proposes the method of so-called *reduction sentences*, which only *conditionally* equate theoretical sentences and protocol sentences. This is a weaker requirement than that of reduction in the sense of the *Aufbau*.

In ‘Two dogmas of empiricism’, Quine proposes another reason why the *Aufbau* reduction project could not succeed. He claims that in a sentence about physical space-time of the form

(5) Quality *q* is at *x*; *y*; *z*; *t*

the expression ‘is at’ remains undefinable from the basic language. We will return to this claim in the next section. It is intimately connected to the question of the definability of ‘here’.

In (1936, p. 464), Carnap does introduce non-indexical reference to times:

(6) On May 6, 1935, at 4 P.M., there is a round table in my room.

but still allows an indexical reference to locations. The discussion of the possibility of reducing such sentences to sentences about possible perceptions concerns the possibility of eliminating the term ‘table’. Carnap also treats ‘my room’ as subject to elimination, but without stressing that the use of the indexical is problematic over and above the use of the ordinary language noun ‘room’. Presumably, the possibility of eliminating indexicals from the physicalistic language was seen as desirable but unproblematic.

I don’t know when the elimination of indexicals was first explicitly raised in the literature. It might have been by Nelson Goodman (1951), in the last chapter of *The Structure of Appearance*. Goodman says that for “formal discourse” the context-dependence (“being not freely repeatable”) of sentences with tense or indexicals (what Goodman calls “indicator words”) is awkward, even though of great practical utility (Goodman 1951, p. 268). He suggests translation as a way out. Thus, (7a) is translated into (7b).

(7)
  - Randy is running now
  - Randy runs [tenseless] on October 17, 1948, at noon E.S.T.

He considers an objection:

Against such translations, it is sometimes urged that they do not really convey the content of the originals. Aspoken [(7a)] tells us that the action takes place at the very moment of speaking, while a[spoken (7b)] does not tell us that the action takes place simultaneously with either utterance unless we know in addition that the time of the utterance is October 17, 1948 at 10 P.M., E.S.T. Since—the argument runs—we recognize the tenseless sentence as atranslation of the tensed one only in the light of outside knowledge, we have here no genuine translation at all. But this seems to me no more cogent than would the parallel argument that “L’Angleterre” is not agenuine translation of “England” because we recognize it as atranslation only if we know that L’Angleterre is England.

Goodman’s requirements on acceptable translations is similar to Carnap’s in 1932. I interpret Goodman here as a) taking the correlation between utterances of sentences like (7a) and sentences like (7b) as a sufficient basis for acceptable translation, and as b) taking the translatability as indicating that nothing is semantically lost in moving from (a)-sentences to (b)-sentences.

Neil Wilson (1955) also proposes a sentence format with explicit time reference and a tenseless copula, although the topic of the context here is not expressive completeness, but a discussion in metaphysics.^6^

In *Word and Object* (Quine 1960), by contrast, the elimination of tense and temporal context dependence does concern expressive power. Quine argues that we can replace the tensed copula by a tenseless copula by introducing explicit reference to time (Quine 1960, p. 170). (8a) is transformed into (8b):

(8)
  - I will not do it again.
  - I do not do it after now.

Here ‘do’ is supposed to be tenseless. He follows up by arguing for the elimination of indexicals (“indicator words”) in order to arrive at so-called “eternal sentences”:

An eternal sentence may be expected to be free of indicator words, but there is no bar to its containing names, however parsed (§37), or other ostensively learned terms. Terms present may well have been learned with help of indicator words (Quine 1960, p. 193).

Quine goes on to say that

I find no good reason not to regard every proposition as nameable by applying brackets to one or another eternal sentence (Quine 1960, p. 193).

In effect, Quine here suggests that a language without temporal context dependence can be expressively complete.^7^

However, already the year before, Prior (1959) argues to the contrary. Prior explicitly objects to Wilson’s (1955) proposal to make the copula tenseless by means of explicit time reference. He claims that some tensed sentences simply are not synonymous with any tensed ones:

One says, e.g. “Thank goodness that’s over!”, and not only is this, when said, quite clear without any date appended, but it says something which it is impossible that any use of atenseless copula with adate should convey. It certainly doesn’t mean the same as, e.g. “Thank goodness the date of the conclusion of that thing is Friday, June 15, 1954”, even if it be said then. (Nor, for that matter, does it mean “Thank goodness the conclusion of that thing is contemporaneous with this utterance”. Why should anyone thank goodness for that?) (Prior 1959, p. 17)

Prior makes two contrasts, the first between retaining or eliminating temporal context dependence ((9a) and (9b)) and the second between tense and explicit token-reflectiveness ((9a) and (9c)):

(9)
  - Thank goodness that’s over.
  - Thank goodness the date of the conclusion of that thing is Friday, June 15, 1954.
  - Thank goodness the conclusion of that thing is contemporaneous with this utterance.

It seems clear, by today’s standards, that the first contrast is the more important one: doing without context dependence leads to a loss of expressive power. Tense, or indexical temporal expressions, are related to present *perspective*, which non-context dependent expressions lack.^8^

Prior’s idea is related to the theme in a series of papers in the mid-1960s by Hector-Neri Castañeda, including Castañeda (1966, 1968). He emphasized the difference between knowledge about someone who is in fact oneself, and genuine self-knowledge. He focused on the logical form of the attribution of self-knowledge to others. There is a difference between

(10)
  - The Editor of *Soul* knows that he is a millionaire.
  - The Editor of *Soul* knows that he (himself) is a millianaire.

Castañeda (1968, p. 440). (10a) can be true if the editor of *Soul* knows that the winner of the state lottery is a millionaire, and he is himself the winner of the state lottery, although he does not know that. (10b), by contrast, as it is interpreted by default, can only be true if the Editor of *Soul* has self-knowledge. This is knowledge that he would express by means of the first-person pronoun:

(11) I am a millionaire.

The first-person pronoun is connected with a subjective, first-person perspective on oneself, which sets it apart from any other way referring to oneself.

This theme from Castañeda was taken up by John Perry (especially Perry 1977, 1979), and indeed, with the additions from David Lewis (1979b), taken to its logical conclusion. In his famous thought experiment (Perry 1977, p. 492), he presents the case of the amnesiac Rudolf Lingens, who is lost in the Stanford Library, where he reads, among other things, a biography about himself. Perry stresses that Lingens’s knowledge about himself is not self-knowledge. Only when he can say “*This* is aisle five, floor six, of Main Library, Stanford. *I* am Rudolf Lingens”, does he express self-knowledge.

Perry in fact makes the substantial assumption that a non-indexical language can be expressively complete. Every proposition can be expressed by means of a non-indexical sentence. In a sense, Lingens does know that he is in the Stanford library, for he knows that *Lingens* is in the Stanford library, and the content of that knowledge is the same as the content of the thought that *he* is in the Stanford library.

This is also David Kaplan’s (1989) position. For instance, on Kaplan’s account, the *content* of an utterance by Kaplan of (12a) in Portland on 26 March 1977 is about the same as that of an utterance by anyone at anytime of (12b) (depending only the size of the *here* and the *now*).

(12)
  - I am here now.
  - David Kaplan is in Portland on 26 March 1977.

Kaplan (1989, pp. 508–9). Thus, at the level of *content*, non-indexical language is expressively complete. Of course, this does not hold at the level of *character*, but propositions are contents, not characters.

If non-indexical language is expressively complete, then, in principle, the truth value of any proposition can be learned by reading up on the world in a comprehensive library. The resulting problem, which Perry, Lewis and Kaplan drew attention to, is that there seems to be something left out, some possible knowledge that Lingens lacks. So, it seems on the one hand that he knows everything, and on the other that there is something that he could know but doesn’t.

Perry called beliefs about who or where one is “locating belief” (1979, p. 5), and they have come to be known, after Lewis (1979a, p. 522) as “self-locating” beliefs. The problem of the nature of self-locating beliefs was discussed early on by Perry, by Kaplan (1989), David (1979a), and Stalnaker (1981), and later by many others. Different solutions were proposed.

I am not here going to propose a solution, either to the problem of self-locating beliefs, or to the problem of world construction. On the contrary, my view is that there is no solution to either: Auto-psychological language as well as non-indexical language is expressively *incomplete*. What I shall argue in the following sections is that that the two problems are virtually equivalent. To put it in a nutshell: first-person facts and third-person facts jointly *underdetermine* the *empirical correlations* between facts of the two kinds.^9^

## World-Construction

I shall here consider a partly simplified and partly idealized version of the world construction project, neither identical to Russell’s nor to Carnap’s. It will differ from Russell’s in having an explicit first-person experience basis, rather than a basis of possible sense-data. It will differ from Carnap’s in accepting phenomenal concepts as given, instead of defining them by means of quasi-analysis, and, more importantly, by providing an explicit adequacy condition, again in partial contrast with the method of quasi-analysis.

We assume given an *internal* language *L*_*i*_ and an *external* language *L*_*e*_. The external language is assumed to be a language for talk about ordinary objects in physical space. The internal language is assumed to be a language describing experiences only. We shall frame the task of *reduction* of *L*_*e*_ to *L*_*i*_ as the existence of a recursively specifiable *translation function* *τ* from *L*_*e*_ to *L*_*i*_ that satisfies a certain adequacy condition. We shall first specify the languages.

- (*L*_*i*_) Let the vocabulary of *L*_*i*_ consist of
  i) demonstratives and proper names (referring to individual experiences)
  ii) *n*-place atomic predicates (for arbitrary *n*) (expressing phenomenal properties of and relations between experiences)
  iii) logical connectives
  iv) Quantifiers ∀ and ∃
  v) Variables *x*_1_, *x*_2_, … ranging over individual experiences, and *y*_1_, *y*_2_,… ranging over sets
  vi) the identity sign (‘=’) and the element sign (‘∈’)

The syntax of *L*_*i*_ consists of the ordinary formation rules of languages of first-order logic. In addition, we assume that new expressions can be introduced by means of definitions. Over and above explicit definitions, contextual definitions and implicit definitions are allowed for. We can also allow for ostensive definitions of proper names.^10^ In the case of implicit definitions, the semantic values of implicitly defined expressions belong to the domain of experiences, properties of experience, sets of experience or properties, sets of sets etc. We let the description operator  as well as the set abstraction operator ‘ {⋅ : …}’ be contextually definable in the usual ways.

As for semantics, we assume a subject *S* having experiences, and a semantic function *μ*_*i*_ that assigns individual experiences of *S* as referents to proper names, as well as to pairs of demonstratives and acts of focusing by *S* on an experience. *μ*_*i*_ also assigns phenomenal properties or relations to atomic predicates. Where *d* is a demonstrative and *r* of *L*_*i*_, *Q* is a two-place atomic predicate of *L*_*i*_, *j* an act of focusing by *S*, we have

(13) *μ*_*i*_(*Q*(*r*, *d*), *j*) = 1 iff *μ*_*i*_(*Q*)(*μ*_*i*_(*r*), *μ*_*i*_(*d*, *j*))

That is: the sentence of *L*_*i*_ ‘ *Q*(*r*, *d*)’ is true, with respect to the act of focusing *j* iff *μ*_*i*_(*r*) and what *S* focuses on in *j* stand in the phenomenal relation *μ*_*i*_(*Q*).

The reason for requiring the values of atomic predicates to be properties rather than merely extensions has to do with assertion. Properties are projectible to new cases. Since we want the subject *S* to be able to assert sentences on the basis of understanding what they express, we cannot rely on mere extensions. An assertion is made at a time, and at no time *t* does *S* know extension of atomic predicates at times later than *t*.

With respect to atomic sentences of *L*_*i*_, as well as to propositional logical compounds of atomic sentences, assertibility and truth coincide:

(TA) For any sentence *s* ∈ *L*_*i*_ that is atomic or a logical compound of atomic sentences of *L*_*i*_, and for any time *t*, *s* is true at *t* iff *s* is assertible by *S* at *t*.

The reason for the coincidence of truth and assertibility for *L*_*i*_ is that *S*’s evidence for the truth of a non-quantified sentence *s* is also the truth-maker for *s*. There is no appearance-reality difference for experiences. We assume that *S* has perfect memory but does not know about the future except by means of inductive inference. The coincidence of truth and assertibility does not extend in general to quantified sentences, of course.

Every event occurs at a time. But the subject *S* does not have a primitive reference to time instants or intervals. Rather, such reference must be defined. We assume that the relations or *earlier than* and *partly simultaneous with* are accessible to *S*. We then assume that *S* uses Russell’s method (1914) of defining times as maximal sets of (partly) simultaneous events. Since *L*_*i*_ is equipped to express quantification over sets of events, it is thereby equipped to quantify over times.

Now for the external language, *L*_*e*_.

- *L*_*e*_ Let the vocabulary of *L*_*i*_ consist of
  i) demonstratives and proper names (as referring to physical individuals and events)
  ii) *n*-place atomic predicates (for arbitrary *n*) (expressing properties of and relations between physical individuals and events)
  iii) logical connectives
  iv) Quantifiers ∀ and ∃
  v) Variables *x*_1_, *x*_2_,… ranging over physical individuals and events, and *y*_1_, *y*_2_,… ranging over sets
  vi) the identity sign (‘=’) and the element sign (‘∈’)

We assume that *L*_*e*_ contains prepositions to express spatio-temporal relations, and that it is equipped with a well-defined notation for specifying spatio-temporal locations. In particular, we assume *L*_*e*_ to contain the sentence-form

(14) *@* (*P*, *x*, *y*, *z*, *t*)

whose instances express propositions that the property *P* is instantiated at spatial location *x*, *y*, *z* at time *t*.

We make no further assumption about the semantics of *L*_*e*_. We assume that *S* has an intuitive understanding of *L*_*e*_, and has dispositions to assert its sentences based on evidence.

We turn now to the question of the *reduction* of *L*_*e*_ to *L*_*i*_. We say that *L*_*e*_ is *reducible* to *L*_*i*_ iff there exists a recursively specifiable translation function *τ* from *L*_*e*_ to *L*_*i*_ that satisfies the *adequacy condition* (AQ(*τ*)) stated below. That *τ* is recursively specifiable simply means here that *τ* is specified by a (computable) clause for each atomic expression of *L*_*e*_, and a (computable) clause for each syntactic construction of *L*_*e*_, allowing a computation of the translation *τ*(*s*) in *L*_*i*_ for each sentence *s* of *L*_*e*_.

We are now ready to state the *adequacy* condition on *τ*:

- (AQ(*τ*)) Necessarily, for any sentence *s* of *L*_*e*_, *s* is *assertible* by *S* iff *τ*(*s*) is assertible by *S*.

This condition, of necessary co-assertibility, does require some motivation. Historically, it parallels the goals of the logicist program in the philosophy of mathematics. The goal of logicism was to a) define mathematical concepts from logical concepts, and b) prove the mathematical theorems from logical axioms and rules of inference. The b) part meant, of course, that *accepted* mathematical theorems, proved within accepted mathematics, would be provable from the logical basis. A strong form of logicism (cf. Tennant 2014) maintains that all mathematical truths are logical truths, not only mathematical theorems. However, as an *adequacy condition* on a proposed reduction, only known theorems can be used as a check.^11^

Similarly, we clearly cannot require that any sentence of an external language that is true according to some realist semantics would be translated into a true sentence of the internal language. In particular, if a sentence is true for which there is no observable evidence, or even no observed evidence, no collection of experiences could be a truth maker. The whole point of the reduction project is the prospect that sentences of the external language are true only to the extent that they have truth makers made up of experiences and abstract operations. Thus, if a sentence *s* of the external language is assertible, based on evidence known by the speaker, then, according to the reduction project, the evidence is ultimately experiential cum abstract evidence. *s* should therefore be translated into an assertible sentence of the internal language. We cannot of course require that any sentence of the internal language should be translatable to a sentence of the external language, for not every collection of experiences correspond to an external state of affairs.

Therefore, for a given translation function *τ*′ and a sentence *s* of *L*_*e*_, if *s* is assertible by *S* while *τ*′(*s*) is not, or vice versa, *τ*′ is not an adequate translation function. If there is *no* adequate translation function from *L*_*e*_ to *L*_*i*_, the reduction project fails.

The adequacy condition is modally strengthened, however. The strenghtening should be understood as requiring that in any possible world where the languages *L*_*i*_ and *L*_*e*_ have the same meaning for *S* as in the actual world, assertibility between any sentence *s* of *L*_*e*_ and its translation *τ*(*s*) coincide.^12^ The idea behind the modal strengthening is simple: if the sentences of *L*_*e*_ are *semantically* reducible to the sentences of *L*_*i*_, then it cannot depend on contingent non-semantic facts whether assertibility is preserved by the translation. The non-semantic facts that vary between worlds are facts about what experiences *S* has, and the distribution of physical objects and properties. If we have a sentence of *s* of *L*_*e*_ that coincides in assertibility with *τ*(*s*) in world *w*_1_, but not in a world *w*_2_, then the evidence for asserting *s* in *w*_1_ is *not* exhausted by the truth-maker for *τ*(*s*) in *w*_1_ either: there is gap in assertibility conditions between the sentences that is bridged by contingent facts in *w*_1_ but not in *w*_2_. As we shall see, this is exactly what happens.

Historically, the general adequacy conditions on reduction have not played aprominent role. Russell speaks at the end of Lecture III, in Russell (1914, p. 44), about accommodating the data by hypothetical construction:

Our hypothetical construction […] shows that the account of the world given by common sense and physical science can be interpreted in away which is logically unobjectionable, and finds aplace for all the data, both hard and soft. It is this hypothetical construction, with its reconciliation of psychology and physics, which is the chief outcome of our discussion.

The distinction between hard and soft data (Russell 1914, p. 34) distinguishes the hard data which will “resist the solvent influence of critical reflection” from the soft data “which, under the operation of this process, become to our minds more or less doubtful”. Everyday beliefs about the physical world would then contribute to what counts as soft data. The quote above can then be understood as imposing the requirement that such beliefs come out justified by way of the construction. This in turn can be recast as a requirement on the translation function as currently conceived.

In addition, Russell also appeals to empirical beliefs, namely the accepted physical laws, for selecting those classes of *aspects* which constitute *things*:

Things are those series of aspects which obey the laws of physics. That such series exist is an empirical fact, which constitutes the verifiability of physics (Russell 1914, p. 49).

Again in terms of the current framework: we select a translation function *τ* from sentences of the external language that contain reference to things, to the sentences of the internal language, such that the translations of sentences expressing accepted laws of physics are assertible on the basis of experience/sense data.

The issue of the adequacy condition in connection with Carnap’s project is rather tricky. In his explanation of what aconstruction is, Carnap says

According to the explanation given above, if an object *a* is reducible to objects *b*, *c*, then all statements about *a* can be transformed into statements about *b* and *c*. To reduce *a* to *b*, *c* or to construct *a* out of *b*, *c* means to produce ageneral rule that indicates for each individual case how astatement about *a* must be transformed in order to yield astatement about *b*, *c*. This rule of translation we call aconstruction rule or *constructional definition* (it has the form of adefinition; cf. §38). (Carnap 1928, p. 6)

This passage by itself does not impose any adequacy condition on the general rule (corresponding to our translation function) itself. However, immediately before this passage Carnap provides the example of reducing *fractions* to pairs of natural number by reducing statements about the former to statements about the latter. In the example, (15a) reduces to (15b):

(15)
  - 3/7 > 2/5
  - For any natural numbers *x* and *y*, if 7*x* = 5*y*, then 3*x* > 2*y*.

The general rule exemplified here is of course adequate: it does transform true/assertible inequality statements about fractions to true/assertible quantified statements about pairs of natural numbers.

When discussing the relation between original expressions and their constructional definitions in §50 (Carnap 1928, pp. 83–4), Carnap claims that the constructed expression (translation) frequently has a different *meaning*, but that the *extension* (in the case of sentences, truth value), is preserved. Carnap says that the constructional method is concerned solely with the *logical value* of a sentence, not with its *epistemic* value. This suggests opting for an adequacy condition in terms of truth value, leaving assertibility out. However, in the preface to the second edition of the *Aufbau*, Carnap states that coextensiveness was too weak a requirement (Carnap 1928, p. ix), for the coextensiveness must not be *accidental*, but *necessary*. Then assertibility, and even necessary co-assertibility, again becomes relevant (which is not to say that this is the notion Carnap had in mind).

The situation is complicated, however, by Carnap’s idea of *quasi-analysis*. Ordinary analysis specify the constituents of complex objects (Carnap 1928, p. 112). Quasi-analysis, by contrast, is applied to *unanalyzable units*, and construct “quasi-characteristics” or “quasi-constituents” that have no independent existence (Carnap 1928, p. 114). It seems therefore that while ordinary analysis can be *correct* or *incorrect*, this does not really hold of quasi-analysis. In the case of quasi-analysis, it would seem, there is no independent standard of correctness (except for consistency).

This is not, however, Carnap’s real standpoint. He does distinguish between desired and undesired results:

We have mentioned here and earlier (§72) that the application of the method of quasi analysis leads to the desired result only if special “unfavorable conditions” do not obtain. These unfavorable conditions could consist, for example, in the fact that certain ^*p*^qualities ^*p*^ always or frequently occur together with certain others. This would lead to irregularities in the derivation of ^*c*^quality classes ^*c*^ and later on in the division into ^*c*^sensory classes ^*c*^ and in the ^*c*^Sim-order ^*c*^ within the sensory classes. However, amore detailed investigation,which we have to omit for lack of space, shows that these interferences in the concept formation through quasi analysis can occur only if circumstances are present under which the real process of cognition, namely, the intuitive quasi analysis which is carried out in real life, would also not lead to normal results (Carnap 1928, p. 133).

Thus, not only can quasi analysis lead to undesired results. Carnap also assumes that it is legitimate to regard our ordinary real-world conceptions and beliefs as resulting from a quasi-analysis that is tacitly carried out “in real life”. Hence, whatever properties we have identified in ordinary cognitive practice, and whatever justified beliefs we have arrived at in it, it should be possible to arrive at the same results through an explicitly reconstructed quasi-analysis.^13^

All in all, I think (AQ (*τ*)) is in itself justified, and well motivated from the historical point of view.^14^

## The World-Construction Problem

I shall consider two kinds of problem for the world construction project, Type 1 and Type 2 problems. The former will be discussed rather briefly, and set aside as less important. We shall then focus on Type 2 problems.

Type 1 problems have to do generally with the relation between theory and observation. Some are exemplified by Carnap (1936, pp. 464–66). He notes that there is no finite set of protocol sentences that would correspond to a simple sentence about a physical thing such as (6):

(6) On May 6, 1935, at 4 P.M., there is a round table in my room.

The reduction of (6) would include (as a first step) sentences of the kind

(16) If on May … somebody is in my room and looks in such and such direction, he has a visual impression of such and such a kind.

The first problem Carnap draws attention to is that no finite set of such sentences (which would also have to take account of other sense modalities) is equivalent with (6). Although incontestably true, this is only an example of the general theory-observation relation. The theory entails the observation sentences, but no finite collection of observation sentences jointly entail the theory. This holds for theories stated in an external language in relation to observation sentences (or other non-theoretical atomic sentences) in the external language itself, and likewise for theories stated in an internal language in relation to atomic sentences of the same language. It has nothing in particular to do with the reduction problem. It is exemplified in the problem of induction.

Carnap moves on to point out “a more serious objection”: sentences of the (16) kind come out true if there is neither an observer nor a chair in his room. Again, this is incontestable, but a result of only considering actual observations. Even Quine, who did not in general accept intensional idioms, talked about “all possible observations” in discussing underdetermination (Quine 1975, p. 313).

The theme of underdetermination of theory by observation connects with Carnap’s first problem. The problem of underdetermination, as Quine (1975) presents it, is that two theories can entail exactly the same set of observation conditionals (a conditional where both the antecedent and the consequent are observation sentences; Quine 1975, p. 318). All possible observations together determine which observation conditionals are true. Conversely, the truth values of all observation conditionals fixes what the possible observations are (given that every possible observation can be expressed by an observation sentence). Hence, underdetermination by the set of all observation conditionals is underdetermination by all possible observations.

This leads over to Type 2 problems. Type 2 problems specifically concern the correlation between experience and external facts, translated into the correlation between the internal and the external language. It does not seem implausible that the totality of experience *underdetermines* physical reality, even when we restrict ourselves to *observable* physical reality. The observable physical reality is that part of physical reality about which we are justified to make assertions based only on definitions and observation. Thus, we are not taking into consideration the possibility of underdetermination of physical theory by observations statable in the external language itself. We can then imagine the following.

1. There are two semantically independent sentences *s*_1_ and *s*_2_ of *L*_*e*_ that report the total sequence of facts observed by *S*. Any translation function *τ* from *L*_*e*_ to *L*_*i*_ that satisfies the semi-adequacy condition (AQ + ) is such that *τ*(*s*_1_) = *τ*(*s*_2_).

Here (AQ + ) is the *only if*-part of (AQ(*τ*)):

(AQ+) Necessarily, for any sentence *s* of *L*_*e*_, if *τ*(*s*) is *assertible* by *S*, then *s* is assertible by *S*.

Thus, the assumption that *S* has made a total sequence of observations described by *τ*(*s*_1_) entails that *S* justified in asserting *τ*(*s*_1_). By (AQ + ), he is then justified in asserting *s*_1_. But the same holds of *s*_2_. If *S* has made a total sequence of observations described by *τ*(*s*_2_), he is justified in asserting *τ*(*s*_2_). By (AQ + ), he is then justified in asserting *s*_2_. Since *τ*(*s*_1_) = *τ*(*s*_2_), it follows that the same sequences of experiences/observations that make *S* is justified in asserting *s*_1_ also make *S* justified in asserting *s*_2_.

But this means that (AQ (*τ*)) is not satisfied. For *s*_1_ and *s*_2_ are semantically independent. Hence being justified in asserting *s*_1_ does not entail being justified in asserting *s*_2_, and vice versa. But if *S* would be justified in asserting *s*_1_, *S* would, by (AQ(*τ*)), be justified in asserting *τ*(*s*_1_), and therefore by (UD1), justified in asserting *τ*(*s*_2_), and then by (AQ + ), justified in asserting *s*_2_, contradicting the independence assumption.

What would be examples of asentence-pair such as *s*_1_ and *s*_2_? In ‘Two Dogmas’, Quine makes the following well-known comment about Carnap’s execution of the *Aufbau* project:

[…] it provides no indication, not even the sketchiest, of how astatement of the form ‘Quality *q* is at *x*; *y*; *z*; *t*’ could ever be translated into Carnap’s initial language of sense data and logic. The connective ‘is at’ remains an added undefined connective; the canons counsel us in its use but not in its elimination (1951, p. 40).

Judging from the *Aufbau* commentary by Alan Richardson (2008), the observation is original with Quine, and also correct. It is one thing, however, to point out that Carnap slurs over the reduction of the ‘is at’ idiom and the spatio-temporal location vocabulary, and another to show that the reduction is impossible. Quine does the former, not the latter. Is there any reason to think that it is impossible?

We find one, I think, if we make use of an idea suggested by Max Black (1952, p. 156) and later also made use of Peter Strawson (1959, p. 20). The idea is that of a possible symmetric universe, with two parts that are qualitatively identical. We can think of it as two similar spheres, as did Black, or as one sphere with two similar halves. I shall here assume the latter, a sphere with two halves, A and B, that are qualitatively identical. In such a universe, a perceiving subject would get exactly the same sequence of experiences whether he starts out in A or in the exactly similar corresponding part in B. The argument, then, is based on the idea that any sequence of experiences *underdetermines* the physical location, since whatever the location of the experiencing subject is, he *could have had* those very experiences (or experiences indistinguishable from them) in the exactly similar but opposite location.

We assume, for *reductio*, that aplausible definition of ‘is at’, and of space coordinates, can be made by *S* in *L*_*i*_. We need not know exactly how, but we can assume it as done along the lines suggested by Russell (1914, pp. 41–2). Russell suggested constructing physical space as asystem of *perspectives*. Each perspective is aview of the universe, whether actually perceived or not (a perceived perspective is a“private world”). Perspectives can be ordered by similarity, and apoint in constructed space is associated with aperspective. Two points are close in constructed space just in case their associated perspectives are *similar*. He suggests adefinition of “here”:

We may define “here” as the place, in perspective space, which is occupied by our private world. Thus we can now understand what is meant by speaking of athing as near to or far from “here”. Athing is near to “here” if the place where it is is near to my private world. We can also understand what is meant by saying that our private world is inside our head; for our private world is aplace in perspective space, and may be part of the place where our head is Russell (1914, p. 42).

Applying Russell’s method straight off in a symmetric universe would have the effect of collapsing the universe into half of it. For every perspective induced from a location in one of the halves would be qualitatively identical to the perspective induced from the corresponding location on the opposite half, and the similarity relations to the respective nearby perspectives on each half would again match each other perfectly. In terms of the similarity of perspectives, then, the duplication of qualitatively similar locations would not be registered. However, we could block this result by adding kinaesthetic experiences, recording change of orientation as well as movement. We would then be able to distinguish between moving across the boundary from A into the opposite half, B, from turning back towards the center of A.

Carnap, in §126 the *Aufbau* (1928, pp. 195–7), somewhat similar to Russell, presents desiderata on the assignment of colours to world-points. For instance, “The color of a visual sensation is assigned to a world point of the corresponding line of view” (p. 196). Carnap does not, however, propose a definition. He comments on this in the preface to the second edition of the *Aufbau* by saying that his procedure is related to the method of introducing concepts by means of *postulates* (I take this to amount to the idea of implicit definition; see note 17 on Leitgeb’s method).

Suppose then that our subject *S*, applying the kinaesthetically modified Russellian method, or any similar method, defines a coordinate system in *L*_*i*_. We assume then that sentences of the form

(14) @ (*P*, *x*, *y*, *z*, *t*)

are definable in *L*_*i*_ (by assumption they occur in *L*_*e*_), as short for “Quality *q* is at space-time location (*x*, *y*, *z*, *t*)”.^15^ And I shall assume a translation function *τ* that maps any sentence of *L*_*e*_ of the form (14) on the syntactically *same* sentence of *L*_*i*_. This means that any numeral in the *x* argument place is mapped on the same numeral in the *x* argument place, and so on. I shall assume two possible worlds *w*_*A*_ and *w*_*B*_. In *w*_*A*_, *S* is initially located at coordinates *x*_*A*_, *y*_*A*_, *z*_*A*_ in sector A. In world *w*_*B*_, by contrast, *S* is initially located in the *corresponding*, qualitatively identical location in sector B, with coordinates *x*_*B*_, *y*_*B*_, *z*_*B*_. Of course, the sentence is an abbreviation of *some* sentence in the primitive vocabulary and syntax of *L*_*i*_.

Assuming that *S* has adequate perceptual abilities, correctly observing the instantiation of qualities. Then, in *w*_*A*_, co-assertibility is perfectly upheld, as far as sentences of the form (14) are concerned. The *L*_*e*_ sentence

(14) @ (*blue*, *x*_1_, *y*_1_, *z*_1_, *t*_1_)

is assertible by *S* on the basis of observation. It is mapped by *τ* on the very same sentence (syntactically) of *L*_*i*_, which is likewise assertible by *S* on the basis of experience.

In world *w*_*B*_, however, *τ* does *not* preserve assertibility. When the sentence (17), as a sentence of *L*_*i*_ becomes assertible by *S*, it is not the *same* sentence in *L*_*e*_ that becomes assertible, but a sentence

(17) @ (*blue*, *x*_2_, *y*_2_, *z*_2_, *t*_1_)

where the space coordinates specify the location in sector B that is qualitatively identical to the location in sector A specified by the space coordinates in (17). Note that the only difference between worlds *w*_*A*_ and *w*_*B*_ is the physical location of *S*. The vocabulary in *L*_*i*_ is defined in exactly the same way in relation to the experiences that *S* undergoes, and these are phenomenally identical in *w*_*A*_ and *w*_*B*_. So the semantics of *L*_*i*_ is the same in the two worlds. Note further that the semantics of *L*_*e*_ is the same as well. The coordinates specify external locations, and the same locations are specified in *L*_*e*_ in *w*_*B*_ as in *w*_*A*_. Therefore, since the co-assertibility of (17) and *τ*(17) is preserved in *w*_*A*_ but not in *w*_*B*_, *τ* does not meet the (AQ(*τ*)) condition.

Does any other translation function meet the (AQ(*τ*)) condition? The alternative translation function *τ*′, which reflects the initial position of *S* in *w*_*B*_ and maps (17) in *L*_*e*_ on (18) in *L*_*i*_ does preserve assertibility in *w*_*B*_. But for the parallel reasons *τ*′ fails to preserve assertibility in *w*_*A*_. And no *other* translation function would preserve assertibility even *within* the two respective worlds. Hence, no translation function satisfies (AQ(*τ*)), and thus the reduction fails.

It must be observed that on the assumption of symmetry between sectors A and B, sentence (17) is true iff sentence (18) is true. The assertibility of (17) (by *S*) will not coincide with the assertibility of (18) (by *S*), because *S* will have evidence either for the one or for the other, depending in *S*’s location. So, in this respect the argument depends on a variation in assertibility that does not match a variation in truth value.

However, on the assumption that *S* is a physical subject, for instance a human being, *S* occupies some space and will make a difference to what is observable by *S*. This will break the symmetry. For instance, should (17) be a sentence reporting a quality that depends on the presence of *S* himself at the location, the assertibility of (17) as a sentence of *L*_*e*_ will neither be assertible nor *true* in *w*_*B*_, for the blueness is reported by (17) to obtain in sector A, while in *w*_*B*_ it only obtains in sector B.

We can, nevertheless, restore complete symmetry by assuming the existence of a physically similar *second* subject, *S*′, who starts out (perhaps having experiences) in the opposite sector, in sector B in world *w*_*A*_ and in sector A in world *w*_*B*_, moving around in exactly the analogous way.^16^

This duplication of observers blocks truth value variation of sentences of the form (14) between *w*_*A*_ and *w*_*B*_. The distribution of qualities is the same in both worlds. Nonetheless, we can add *self-reflective* sentences that will vary in truth value between the two worlds. We need a form of sentence that is different from (14):

(19) @ (*a*,(*x*, *y*, *z*, *t*))

saying that an *object* *a* is at the spatial location (*x*, *y*, *z*) at time *t*. In the external language, this makes reference to a physical object. In the internal language, *a* is a construction of sense data / experience. As a special case of (19), we have

20 @ (I,(*x*, *y*, *z*, *t*))

where ‘I’ is the first-person pronoun. Can the first-person pronoun be defined?

Russell (1914, p. 42) claims that there are two places associated with every aspect of athing, and continues:

Every aspect of athing is amember of two different classes of aspects, namely: (1) the various aspects of the thing, of which at most one appears in any given perspective; (2) the perspective of which the given aspect is amember, *i.e.* that in which the thing has the given aspect. The physicist naturally classifies aspects in the first way, the psychologist in the second. The two places associated with asingle aspect correspond to the two ways of classifying it. We may distinguish the two places as that *at* which, and that *from* which, the aspect appears.

And just before this, Russell says that “[…] our private world is a place in perspective space, and may be part of the place where our head is”. Thus, the subject can define a term referring to his own head or body as the object occupying the place from which aspects of things appear.

Similarly, Carnap (1928, pp. 199–200) suggests the the expression “my body” can be defined as that thing satisfying certain conditions, including constant visual proximity and correlation between experiences of the visual and the tactile sense modalities. And the *self* (or “my mind”) can be defined as the class of “autopsychological states” (Carnap 1928, p. 205).

So let us assume that the bodily self is constructible, and that ‘I’ can be defined to refer to it. Let us also assume that ‘I’ exists in the *external* language as a self-referring expression. Assume further that *τ* maps ‘I’ on itself (syntactically). Also, assume that the ‘@’ predicate is defined for such referring expressions, not just quality terms. Then consider

(21) @ (I, *x*_1_, *y*_1_, *z*_1_, *t*_1_)

In *w*_*A*_, both (21) of *L*_*e*_ and its translation *τ*(21) in *L*_*i*_, syntactically identical to (21), are assertible by *S* and true. In *w*_*B*_, however, (21) in *L*_*i*_ is still assertible and true, while (21) as a sentence of *L*_*e*_, the external language, is not only not assertible, but also false. It is false, because *S* at time *t*_1_ is not at (*x*_1_, *y*_1_, *z*_1_) in sector A but at (*x*_2_, *y*_2_, *z*_2_) in sector B. Hence, the reduction fails, also with respect to truth value underdetermination.

Again, at bottom, the failure depends on the fact that, in a symmetric universe, the totality of experiences underdetermines the physical facts, in particular the fact of the location of the subject *S*. Note that adding the general physical features of the universe, except for the location of *S*, still underdetermines *S*’s location. So knowing these external facts about the universe will not help. Note further that underdetermination still obtains after adding knowledge of the *qualitative* correlation between the environment and the experiences (an environment of such-and-such qualities causes such-and-such experiences). In addition, if we keep the assumption of a second experiencing subject *S*′, even if *S* *knows* that *S* is in sector A while *S*′ is in sector B, this is not enough unless *S* also knows that *he* is *S* rather than *S*′ (which highlights the self-location aspect).^17^

## The Self-Location Problem

In his seminal paper Frege on Demonstratives (Perry 1977), John Perry presents the case of the amnesiacs Lingens, who finds himself in a big library with no recollection of his past, but with access to written information about the world, including information about an amnesiac named ‘Lingens’ who is lost in the Stanford library. If we could add that he has access to a true and complete–non-indexical–theory of the world, Lingens could infer that *he* is Lingens, by first-person knowledge of his memory loss and that he is in a big library, together with the library knowledge that there is exactly one person in the world that is in such a situation.

This possibility was eliminated in David Lewis’s (1979a, p. 535) extension of the thought experiment, where he adds a second amnesiac, in a qualitatively identical situation, but located at the Harvard library. That is Lauben.^18^ The libraries are excellent, in fact in a certain respect complete. Every objective proposition that has been made true at the time can be learned from the books available in these libraries.

By ‘objective proposition’ is meant propositions that are not essentially *indexical*. That is, every objective proposition can be *expressed* in wholly non-indexical vocabulary. When David Kaplan utters ‘I am here now’ in Portland on 26 March 1977, if what he expresses is an objective proposition, then that proposition can equally well be expressed by ‘David Kaplan is in Portland on 26 March 1977’. The former expression is an indexical way of presenting a proposition, and the latter expression a non-indexical way of presenting the very same proposition. Both Lingens and Lauben have access to records of every objective proposition that has been made true at the time, but only presented in non-indexical ways. This means, among other things, that Lingens will not find a sign saying ‘This is Stanford library’, giving him the indexical information that he is in Stanford library.

Because of knowing all objective propositions true at the time, both Lingens and Lauben know that there are two amnesiacs, named ‘Lingens’ and ‘Lauben’ in the Stanford and Harvard library, respectively, that read the books and learn about themselves.^19^ The epistemic problem for Lingens (and Lauben) is that this information is not enough for determining where he is, in Stanford or in Harvard. And since he knows that Lingens is in Stanford and Lauben in Harvard, he knows that he is Lingens if he is in Stanford and Lauben if he is in Harvard, he also does not know which of them he is. He can know, by combining knowledge from reading and knowledge from experience (perception and introspection), and applying Leibniz’s law, that he is distinct from every one other than Lingens and Lauben, but not which of the two he is. All his encyclopedia knowledge and all his experience put together is compatible with both alternatives.

Now there is a question in which sense there is something that Lingens does not know, and that question is also the main topic of Perry’s paper. If all propositions are objective propositions then there is no true *proposition* that Lingens does not know is true. He considers the sentence

(22) I am Lingens

and comes to the conclusion that he does not know it’s truth value. But if there are only objective propositions, then what is expressed by (22) is a proposition whose truth value he already does know. For instance, as is considered by Perry, if the contribution of ‘I’ to the proposition expressed is just the speaker of the sentence on the occasion, and if the contribution of the name ‘Lingens’ is just the referent, Lingens, then (22) expresses the same proposition as

(23) Lingens is Lingens

which, of course, is known to be true by both Lingens and Lauben. So there is a question in what sense Lingens does not know that he is Lingens. Similarly, there is a question in which sense he can *believe* that *he* is Lingens, or Lauben, or in Stanford, or in Harvard. This is the problem of self-locating beliefs.^20^

Various solutions have been offered (belief states, centered-worlds propositions, reflexive propositions), but our task here is to characterize the problem in a semantic way. Thus, we can assume two languages, *L*_*s*_ (the subjective language) and *L*_*o*_ (the objective language). The vocabulary of the two languages are as follows:

- *L*_*s*_ *L*_*s*_ contains
  i) indexicals: *I*, *you*, *here*, *now* …, and demonstratives
  ii) nouns, verbs, and adjectives that can be applied on the basis of observation
  iii) prepositions
  vi) logical connectives, quantifiers, variables

In addition, we allow *L*_*s*_ to be *extended* by the introduction of new proper names, defined either from the original vocabulary or by means of ostensive definitions.

- *L*_*o*_ *L*_*o*_ contains
  i) Proper names, non-indexical terms for locations and times
  ii) nouns, verbs, and adjectives that do not take contextual arguments
  iii) prepositions
  vi) logical connectives, quantifiers, variables

Both languages have standard grammatical constructions to form simple and complex sentences.

By the thought experiment, Lingens’s experiences, together with all (objective) facts underdetermine his location, i.e. underdetermine at which location the experiences are taking place. The assumption that Lingens *knows* all objective propositions but does not know where he is, is a dramatic way of bringing this out. We could also dispense with the appeal to amnesia.^21^

That there is a sense in which Lingens does not know where he is, comes out in the fact that, as a sentence of ordinary English,

(24) I am in Stanford.

is not assertible by Lingens, although true. We can recast this in terms of considering the possibility of a mapping *ν* from *L*_*s*_ to *L*_*o*_, analogous to *τ*. This is the proper mapping direction, since we are considering the question whether *L*_*o*_ is expressively complete: are there sentences of *L*_*s*_ that cannot adequately be translated into *L*_*o*_?

We then need an adequacy condition on *ν* corresponding to (AQ(*τ*)), that *ν* preserves assertibility:

- AQ(*ν*) Necessarily, for any sentence *s* of *L*_*s*_, *s* is assertible by Lingens iff *ν*(*s*) in *L*_*o*_ is assertible by Lingens.

We now consider two worlds, *w*_*S*_, the actual world of the story, where Lingens is in Stanford, and *w*_*H*_, the non-actual world where Lingens is in Harvard and Lauben in Stanford, and the other assumptions are changed as needed, *mutatis mutandis*. We can now contrast

(25) a. I am here.
  b. Lingens is in Stanford.

(25a) is a sentence of *L*_*s*_ and (25b) a sentence of *L*_*o*_. Clearly, (25a) is assertible by Lingens in both *w*_*S*_ and *w*_*H*_, while (25b) is assertible by Lingens in *w*_*S*_ but not in *w*_*H*_. Hence, a translation function *ν* that maps ‘I’ on ‘Lingens’ and ‘here’ on ‘Stanford’ does not satisfy (AQ(*v*)).

This is of course unsurprising in light of the standard difference between indexical and non-indexical sentences, but corresponds well to the situation of the Carnapian subject *S* with respect to the sentence (21). We might even consider a sentence-pair like

(26) a. Blue spot at position *z* in this library.
  b. Blue spot at position *z* in Stanford library.

(26a) is a sentence of *L*_*s*_ and (26b) a sentence of *L*_*o*_. (26a) is assertible by Lingens on the basis of observation, in both *w*_*S*_ and *w*_*H*_. If the spot emerges at a time after the period covered by the books in the library, then (26b) is not assertible in *w*_*H*_, even if true (since Lingens has no evidence for its truth). This corresponds well to the situation of *S* with respect to (17) and (18). Of course, if the blue spot *is* described by the library books, then both sentences are assertible by Lingens in both worlds (for there must also be a recorded blue spot in the Harvard library.)

These differences between *L*_*s*_ and *L*_*o*_ depend crucially on the difference between indexical and non-indexical language. It may seem, however, that we could bridge this difference by allowing Lingens access to *new* proper names that he can define by ostension. Thus, let Lingens introduce the names ‘Lingens*’ and ‘Stanford*’ as follows:

(27) a. Let ‘Lingens*’ be a name of me.
  b. Let ‘Stanford*’ be a name of this place (here).

In virtue of these stipulations, the *L*_*s*_ sentence

(28) Lingens* is in Stanford*

is assertible in *w*_*S*_, just as (25a) is. Since the semantic effect will be that ‘Lingens*’ refers to Lingens and ‘Stanford*’ to Stanford, (28), as defined in *w*_*S*_, will have the same truth value as (25b), both in *w*_*S*_ and in *w*_*H*_.^22^ On the other hand, in *w*_*H*_, the stipulations are carried out under the conditions that obtain in *that* world. This means, that in *w*_*H*_, ‘Lingens*’ refers to Lingens, but ‘Stanford*’, as defined in *w*_*H*_, refers to Harvard. So defined, (28) is true in *w*_*H*_ and false in *w*_*S*_. Nevertheless, (28) is *assertible* by Lingens in both *w*_*S*_ and *w*_*H*_, just like (25a).

Although it might seem that the introduction of new proper names in *L*_*s*_ bridges the difference between the two languages, a moment’s reflection shows that this is not really the case. For there is no mapping *ν* from *L*_*s*_ to *L*_*o*_ that preserves meaning and co-assertibility across the two worlds. The translation *ν* that maps ‘Lingens*’ on ‘Lingens’ and ‘Stanford*’ on ‘Stanford’ yields both co-assertibility and co-extension between (28) and (25b) in *w*_*S*_, but it does not preserve extension in *w*_*H*_. For in *w*_*H*_, ‘Stanford*’ is made to refer to Harvard, while *ν*(‘Stanford*’)=‘Stanford’, still refers to Stanford. Likewise, the mapping *ν*′ that maps ‘Stanford*’ on ‘Harvard’, preserves extension in *w*_*H*_ but not in *w*_*S*_. So, the (AQ(*v*)) condition is not met by any mapping from *L*_*s*_ to *L*_*o*_.

The result in this case is not that there are mappings between *L*_*s*_ and *L*_*o*_ *w*_*S*_ and *w*_*H*_ that preserve meaning but none that also preserves co-assertibility across worlds. Rather in this case, there is no mapping that even preserves meaning across the two worlds. This results from the assumption that there is a direct option of introducing proper names in *L*_*s*_ by stipulation. There is no counterpart in the world-construction case to this option.

Still, we can make use in a slightly different way of this option. It is easy to see that there is a difference in the contribution to assertibility for Lingens of the two names ‘Lingens’ and ‘Lingens*’. For if they were the same, the two names would be intersubstitutable in all extensional use contexts, and the name ‘Lingens*’ could be *added* the *objective* language, *L*_*o*_ without changing the expressive power. However, the sentence

(29) Lingens* is in Stanford.

is clearly *not* assertible in *w*_*S*_ by Lingens, in contrast to (25b). If it were, given how the name has been defined, Lingens *would* know that he is in Stanford. This outcome again depends on the fact that it is underdetermined by the book facts and the experiences where the experiences are taking place.

The upshot is that the objective, non-indexical language, is expressively *incomplete*, since there is no adequate translation from the indexical to the non-indexical language.^23^

## Conclusion

The main claim in this paper is that world-construction problem that is generated by failure of reducing space-time location vocabulary is at bottom the same as the problem of self-locating beliefs. We can regard the two problems as problems concerning expressive completeness, in case of the world-construction problem of the *internal language*, and in case of the self-location problem, of the *objective language*.

The reason why completeness fails in both cases is the same, the underdetermination by experiences and objective facts of the correlation between them. More specifically, the total record of experiences together with the total record of objective facts jointly underdetermine the correlation of experiences with objective facts about the location of the observer. Experience facts can be kept constant between two worlds while the objective location varies. That is why there is no adequate, and hence world-independent, translation function from the internal to the external language in the world-construction case, and why here is no adequate translation function from the objective language to the indexical language in the self-location case.

It therefore also turns out that the there is a close parallel between on the one hand the difference between internal experience-language and external language and on the other and the difference between indexical language and non-indexical language. In both cases the problem can be set out in terms of the failure of translations that preserve co-assertibility. The common factor is that both experience language and external *indexical* language can be applied to what is immediately observed, without relying on any background knowledge of the external world.^24^

The two reductions fail because of the same underdetermination. However, this is not enough to show that the two reductions are equivalent in a *stronger* sense: that there is an adequate translation function in the one case iff there is an adequate translation function in the other case. For one reduction could fail for *another* reason that might *not* also make the other reduction fail. This possibility remains to be investigated. The upshot of the present paper is only that there is one basic underdetermination that make both fail, and that they are equivalent in at least this weaker sense.

## Footnotes

1 There may also be *objective internal* facts, e.g. facts described in third-person vocabulary concerning mental states (*John is thinking about vacation*), as well as *subjective external* facts, e.g. physical facts described from a first-person perspective (*my pants are on fire*).

2 It should be noted that the question of expressive completeness is not formally equivalent with the question of reduction. For it may be that everything expressible in *L* is expressible in *L*′, although there is no *recursively definable* translation function from *L* to *L*′. Cf. next section. In the cases under consideration, however, it is because of the failure of expressive completeness that adequate recursive translation functions don’t exist.

3 Carnap was in fact aware of the problem already in the *Aufbau*, and in the end tried to eliminate the one basic auto-psychological concept by means of existential generalization over relations, in order that nothing purely subjective should remain in the system of definitions. Cf. (Carnap 1928, pp. 234–38).

4 I assume here that a reduction of a physicalistic language to an auto-psychological language would proceed via the reduction of theoretical sentences to protocol sentence. On this assumption the failure of the latter reduction has a indirect bearing on the Aufbau reduction project.

5 It seems that we can get around the problem of non-equivalence by using counterfactuals: “If *x* *were to* be put into water, it *would* dissolve”. The problem with this solution is that the part of the truth-conditions of the counterfactual that go beyond the truth-conditions of (4b) are not *testable*. Any testing that is actually known is testing in the actual world, so you only test the counterfactual when you also test (4b). Thus, if you require testability, as Carnap did in 1936, the counterfactual does not add anything to (4b).

6 Wilson wants to introduce *substance*-talk where properties are ascribed to individuals relative to times, to replace the ascribing of properties to space-time points, as had been proposed earlier by Quine (1950).

7 This holds strictly only for scientifically acceptable language. When moving beyond this to belief reports, there are no context independent standards of correctness, according to Quine. In such cases, not even eternalizing the sentence in the belief context is sufficient for eliminating context dependence. Cf. (Quine 1960, p. 218).

8 Basically, Prior’s objection is the same as the one Goodman dismisses, aside from the evaluative component. We might psychologize Prior’s statement into

(i) Prior is happy that that’s over.

From Quine’s point of view, tense or context dependence might not be eliminable here, but this would be an instance of psychological talk where that cannot be required.

9 David Chalmers’s recent contribution to the world construction area (Chalmers 2012) falls outside the present considerations. The reason is that Chalmers includes in the basis of his world construction what he calls the *PQTI* facts: physics, qualia, that’s all-facts, and indexical facts (first mentioned on p. 22.). Hence, Chalmers’s basis already includes types of fact that will here be argued to be not reducible to each other. See also note 11.

10 Strictly speaking, we should then need to take extensions of *L*_*i*_ into account, where new names are added. I shall pass over this complication, and assume that we can talk of the same language throughout.

11 Should we perhaps consider alternative ways of strengthening co-assertibility, with appeal to *analyticity*, or *a priority*, as discussed in chapter 1 of Chalmers (2012)? Analyticity is immediately ruled out unless it holds that an analysis of the *meanings* of the expressions of the external language already shows that they are expressible in terms of the internal language, which is completely implausible. And to the extent that analyticity entails necessity, if the necessity strengthening fails, so does anyway the analyticity strengthening. The a priority strengthening works in the mathematical case, but only because a) the Peano axioms are derivable from the logicist base, and b), a mathematical sentence *s* is a logical consequence of mathematical sentences *s*_1_,…, *s*_*n*_ iff the logicist translation of *s* is a consequence of the logicist translations of *s*_1_,…, *s*_*n*_. This has no counterpart in the empirical case we are concerned with here. Note that Chalmers’s strategy in his (2012) is to keep that reduction base rich enough to allow what he calls *a priori scrutability* (Chalmers 2012, p. 6), which is very different from selecting it by the independent criteria employed by Russell or Carnap. Note for instance that Chalmers takes Quine’s ‘is at’ problem to be solved by “allowing spatiotemporal expressions into the basic vocabulary (Chalmers 2012, p. 9)”.

12 Of course, the translation function itself does not vary between worlds. Rather, the translation function has to satisfy a modal condition.

13 I therefore completely agree with Hannes Leitgeb (2007, p. 184) when he writes “While similarity is prior to properties or qualities on the level of the phenomenalistic constitution system, properties or qualities are prior to similarity on the level of Carnap’s extrasystematic description of the phenomenalistic constitution system. When Carnap discusses the intended results of quasianalysis (cf. §81, 72) and distinguishes them from unintended ones that might occur under unfavourable circumstances, he takes the extrasystematic standpoint.”

14 Leitgeb (2011, p. 268), in his new *Aufbau*-like system, requires that the translation (from a scientific sentence to the sentence constructed from logical vocabulary on terms for experiences) preserves *empirical content*, although not necessarily *extension*. This seems related in spirit to the present proposal of preserving assertibility. Since Leitgeb leaves the notion of empirical content undefined, a precise comparison between his notion of adequacy and the present one cannot be made. However, in view of the underdetermination problem to be set out in the next section, we can say the following: if assertibility tracks empirical content, and if Leitgeb’s new *Aufbau* system succeeds in *preserving* empirical content by the proposed translation, then the truth conditions of sentences assigning colours to space-time points (of the form (15a)), are *not* reducible to their empirical content.

15 Since we are now dealing with the coordinates on the surface of a sphere we could make do with two space coordinates, but I’ll stick with three.

16 This makes the analogy with self-location problem even better.

17 I shall make some brief remarks about the failure result. Firstly, from Carnap’s own point of view, it could be claimed that the reduction will indeed not work in a symmetric universe, because then circumstances are “unfavourable” (see the quote above from Carnap (1928), §§, 70, 72, 81), but will work in an asymmetric universe, and this is good enough. It is indeed good enough if we lower the requirements on what we regard as proper translation, i.e. as a meaning-preserving mapping. See the remarks on Carnap (1932) and on Goodman (1951) above. Secondly, Thomas Mormann (2004) proposes a way (similar in spirit to Russell’s method) of answering Quine’s objection to Carnap by defining a geometric structure directly over experiences. This allows a definition of ‘is at’. It does not eliminate the underdetermination, however. Thirdly, Leitgeb (2011, pp. 289–96), proposes to treat ‘*col* ’, used as a name of the function that assigns colours to space-time points, as implicitly defined by the desiderata provided by Carnap in Section 126 of the *Aufbau*. ‘*col* ’ corresponds to the relational predicate ‘@’ used here. Leitgeb suggests formalizing Carnap’s desiderata as axioms, collectively abbreviated ‘ *A*’. Thus ‘ *A*[*col*]’ implicitly defines ‘*col*’ (Leitgeb 2011, pp. 290-1). Sentences that use ‘*col* ’, of the form ‘*B*[*col* ]’ can then be interpreted by means of this definition. Leitgeb suggests three ways, where the first is by means of a Ramsey sentence: ‘*B*[*col* ]’ is interpreted as ‘ ∃*x*(*A*[*x*] ∧ *B*[*x*])’. The second uses Lewis’s uniqueness condition, and the third Hilbert’s *𝜖* operator. Of course, the reduction project here requires the semantic values of ‘*col* ’ (witnesses of the Ramsey sentence) to be auto-psychological properties, not functions that actually map colours on space-time points (which would be question-begging). But this again makes the definition open to the underdetermination problem.

18 Lewis calls him ‘The Widener amnesiac’. Who first started using ‘Lauben’ as the name, I have not found out.

19 We should assume that they can’t check predictions like ‘At midnight Lingens jumps up and down while Lauben scratches his head’ and then determine who they are by a combination of decision and self-observation.

20 Cappelen and Dever (2013, pp. 100–2) argue that there is nothing special about the *de se* over and above ordinary Fregean problems of coreference. They complain that the problem depends on the coarseness of grain of of possible-worlds account of content. This is not correct, however. As is pointed out by Perry (1979, p. 7), whatever *α* is, a subject might fail to realize the *he* is *α*. This does not depend on grain. We can let Lingens know all objective propositions with a finer grain than that of possible-worlds propositions; the problem with the apparent lack of indexical knowledge arises anyway. Indeed, as Frege himself pointed out, “everyone is presented to himself in a special and primitive way, in which he is presented to no one else” (Frege 1918, p. 333).

21 Lewis’s story about the two gods in Lewis David (1979b) does not appeal to amnesia, but as many have pointed out, the thought experiment itself is problematic, and perhaps not coherent. Kaplan (1989, p. 531) presents the analogous case of Castor and Pollux, two identical twins, raised under qualitatively identical conditions, getting qualitatively identical stimuli (analogous to *S* and *S*′ in the symmetric universe).

22 We assume here that proper names are rigid designators, or directly referring, to keep the discussion in line with Perry’s.

23 More precisely, the objective language is incomplete by the translation co-assertibility condition. By Kaplan’s semantics, and by Perry’s assumptions, as indicated above, the objective language is semantically complete in the sense of able to express the same propositional contents as the indexical language. I leave to another occasion to argue for the condition and against Kaplan’s semantics.

24 The option of introducing new proper names by ostensive definition in the self-location case does not correspond to anything in the world-construction case. It alters the format of the failure to preserve co-assertibility.
